# International randomised controlled trial for the treatment of newly diagnosed EWING sarcoma family of tumours – EURO EWING 2012 Protocol

**DOI:** 10.1186/s13063-019-4026-8

**Published:** 2020-01-17

**Authors:** Jennifer Anderton, Veronica Moroz, Perrine Marec-Bérard, Nathalie Gaspar, Valerie Laurence, Javier Martín-Broto, Ana Sastre, Hans Gelderblom, Cormac Owens, Sophie Kaiser, Melissa Fernández-Pinto, Nicola Fenwick, Abigail Evans, Sandra Strauss, Jeremy Whelan, Keith Wheatley, Bernadette Brennan

**Affiliations:** 1 Cancer Research UK Clinical Trials Unit, University of Birmingham, Mindelsohn Way, Birmingham, B15 2TT UK; 2 Centre Léon Bérard, 28 rue Laënnec, 69373 Lyon cedex 08, France; 3 Société Française de Lutte contre les Cancers et Leucémies de l’Enfant et de l’Adolescent (SFCE), 16 boulevard de Bulgarie, 35203 Rennes, France; 4 Groupe Sarcome Français - Groupe d’Etude des Sarcome Osseux (GSF-GETO), 28 rue Laënnec, 69373 Lyon cedex 08, France; 5 Gustave Roussy Cancer Campus, 114 rue Édouard-Vaillant, 94805 Villejuif, France; 6 Institut Curie, 26 Rue d’Ulm, 75005 Paris, France; 7 Institute of Biomedicine of Sevilla (IBIS, HUVR, CSIC, Universidad de Sevilla), Avda. Manuel Siurot, 41013 Sevilla, Spain; 8 Hospital Universitario La Paz, 261 Paseo de la Castellana, 28046 Madrid, Spain; 9 European Organisation for Research and Treatment of Cancer (EORTC), Avenue Mounier 83, B-1200 Brussels, Belgium; 10 Our Lady’s Children’s Hospital, Cooley Rd, Dublin D12 N512, Ireland; 11 Grupo Español de Investigación en Sarcomas (GEIS), Diego de León St, 47th 28006 Madrid, Spain; 12 University College London, Gower Street, London, WC1E 6BT UK; 13 University College London Hospitals NHS Foundation Trust, 250 Euston Road, London, NW1 2PG UK; 14 Royal Manchester Children’s Hospital, Oxford road, Manchester, M13 9WL UK; 15 University Hospital Virgen del Rocio, Av. Manuel Siurot, 41013 Seville, Spain

**Keywords:** Ewing sarcoma family of tumours, Randomised controlled trial

## Abstract

**Background:**

Although there have been multiple randomised trials in newly diagnosed Ewing sarcoma family of tumours (ESFT) and these have been conducted over many years and involved many international cooperative groups, the outcomes for all stages of disease have plateaued. Internationally, the standard treatment of ESFT is not defined, and there is a need to add new agents other than conventional chemotherapy to improve outcomes. This trial will compare two different induction/consolidation chemotherapy regimens: (1) vincristine, ifosfamide, doxorubicin and etoposide (VIDE) induction and vincristine, actinomycin D, ifosfamide or cyclophosphamide, or busulfan and mephalan (VAI/VAC/BuMel) consolidation and (2) vincristine, doxorubicin, cyclophosphamide, ifosfamide and etoposide (VDC/IE) induction and ifosfamide and etoposide, vincristine and cyclophosphamide, vincristine, actinomycin D and ifosfamide, or busulfan and mephalan (IE/VC/VAI/BuMel) consolidation (randomisation 1, or R1). A second randomisation (R2) will determine whether the addition of zoledronic acid to consolidation chemotherapy, as assigned at R1, is associated with improved clinical outcome.

**Methods:**

EURO EWING 2012 is an international, multicentre, phase III, open-label randomised controlled trial. There are two randomisations: R1 and R2. Patients are randomly assigned at two different time points: at entry to the trial (R1) and following local control therapy (R2). The primary outcome measure is event-free survival. The secondary outcome measures include overall survival, adverse events and toxicity, histological response of the primary tumour, response of the primary tumour, regional lymph nodes or metastases (or both), and achievement of local control at the end of treatment.

**Discussion:**

This study will establish which is the “standard regimen” of chemotherapy, taking into account both clinical outcomes and toxicity. This will form the chemotherapy backbone for future interventional studies where we may want to add new targeted agents. It will also determine the role of zoledronic acid in conjunction with the separate EE2008 trial. Any trial in ESFT needs to take into account the rarity of the tumour and consider that international cooperation is needed to provide answers in a timely manner.

**Trial registration:**

Registered with EudraCT number 2012-002107-17 on 26 February 2012. Registered with ISRCTN number 54540667 on 4 November 2013.

## Background and Rationale

The Ewing sarcoma family of tumours (ESFT) usually arise in skeletal sites in children and young people and consist of small round malignant cells that may exhibit varying degrees of neural differentiation. ESFT are characterised by a re-arrangement involving chromosome 22, and 11;22 translocation is detectable in more than 95% of cases [1–7]. The gene rearrangement results in the production of a transcription factor (in the majority, EWS-FLI1 transcription).

Most ESFT arise in bony sites. Staging procedures identify about 30% of patients with detectable metastases at diagnosis. Since chemotherapy was introduced routinely in the 1970s, cure rates have dramatically improved. With current multimodal programmes, including combination chemotherapy, surgery and radiotherapy, the 5-year survival rate for localised ESFT is about 65% with chemotherapy regimens including actinomycin D, doxorubicin, etoposide, cyclophosphamide, vincristine and ifosfamide. Using different doses and schedules of administration, ESFT with lung-only metastases treated with conventional chemotherapy experience a 3-year event-free survival (EFS) of about 30%, while for those patients with disseminated disease the prognosis remains very poor, the EURO-E.W.I.N.G. 99 trial demonstrating overall survival (OS) at 3 years of 29% [8].

Internationally, a single standard chemotherapy treatment for ESFT is not defined. The EURO-E.W.I.N.G. 99 trial employed VIDE induction chemotherapy (six cycles of vincristine, ifosfamide, doxorubicin and etoposide given about every 3 weeks prior to local control), followed by risk-adapted randomised treatment of either vincristine, actinomycin D and ifosfamide or cyclophosphamide (VAI/VAC) as consolidation chemotherapy or high-dose busulfan/melphalan. The toxicity of VIDE induction chemotherapy has been published [9]. In summary, 12% had grade III or IV stomatitis, 3% had cardiac left ventricular dysfunction as determined by fractional shortening, there were five toxic-related deaths out of 851 patients (giving a rate of 0.6%), and grade II, III and IV infections occurred in 40%, 9% and 0.6% respectively. As yet, the data on second malignant neoplasms (SMNs) have not been published. But in the EURO-E.W.I.N.G. 99 trial between 1 September 2001 and 1 September 2005, there were five SMNs (two leukaemias and three solid tumours) in the 462 registered patients with localised disease (Marie-Cécile Le Deley, personal communication).

The other widely used treatment regimen for ESFT, employed mainly in the US, is from the Children’s Oncology Group AEWS0031 trial [10]. In that study, patients with localised ESFT received alternating cycles of vincristine-doxorubicin-cyclophosphamide and ifosfamide-etoposide (VDC/IE) as induction chemotherapy and alternating cycles of ifosfamide-etoposide and vincristine-cyclophosphamide (IE/VC) as consolidation chemotherapy. There was an upfront randomisation to compare 3-weekly cycles of this treatment (standard arm) with 2-weekly cycles (experimental arm). There was significantly superior EFS of 73% in the compressed 2-weekly VDC/IE/VC compared with 65% in the standard arm (*P* = 0.048) and also improved OS: 83% and 77% respectively (*P* = 0.056). This compressed induction regimen has become the standard regimen for localised ESFT in the US. In regard to short-term toxicity, there was one toxic death in the compressed arm B. In arm B, despite compression of the chemotherapy cycles, stomatitis occurred in 3% and colitis or typhlitis in 0.4% of chemotherapy cycles. There were no episodes of cardiac left ventricular dysfunction, and grade III/IV infectious toxicities occurred as follows: febrile neutropenia 7%, infection with grade 3/4 neutropenia 5%, infection without neutropenia 2% and infection (white cell count unknown) 0.3%. Therefore, a randomisation at diagnosis between VIDE and VAI/VAC versus VDC/IE/VC is necessary to establish which is the regimen of choice, taking account of both clinical outcome (EFS and OS) and toxicity.

Bisphosphonates, a group of compounds that inhibit bone resorption, have been used for the treatment of bone metastases in patients with breast cancer, multiple myeloma and prostate cancer [11, 12]*. In vitro* and *in vivo* data have also proven the anti-tumour activity of nitrogen-containing bisphosphonates (N-BPs) against ESFT cells. The N-BP pamidronate inhibits growth in eight different ESFT cell lines via inhibition of the mevalonate pathway [13]. Zhou et al. showed significant inhibition in the development of bone metastases after injection of the bisphosphonate zoledronic acid *in vivo*; N-BPs induced apoptosis and inhibited osseous metastases [14]. Zoledronic acid has a direct inhibitory effect on the growth of ESFT cells *in vitro* which is induced by apoptosis associated with caspase 3 activation and cell cycle arrest in S phase. This effect was enhanced by alkylating agents. In an *in vivo* mouse model, zoledronic acid exerted a strong inhibitory effect on the growth of bone ESFT and little effect on the growth of intramuscularly injected ESFT. When combined with ifosfamide, zoledronic acid exerted synergistic effects in the soft tissue model: its combination with one cycle of ifosfamide resulted in an inhibitory effect similar to three cycles of ifosfamide alone [15].

Although there are no clinical studies of zoledronic acid in ESFT, except for a single case report of a multiple relapsed patient responding to zoledronic acid with third-line chemotherapy, its low toxicity profile with conventional chemotherapy and the growing body of evidence for the use of bisphosphonates for the treatment of bone metastases in other cancers described above provide ample justification to examine the value of zoledronic acid in a clinical trial.

Although ESFT are the second commonest malignant bone tumour in children, adolescents and young adults, they remain rare tumours (fewer than 70 cases per year in the UK) and hence any randomised trials must be international. The EURO EWING Consortium (EEC) is a partnership of specialists in 15 European countries working together to improve the outcome in ESFT. The activities of the EEC are funded by the European Union for five years and include two clinical trials, translational research and the strengthening of both patient and public involvement and the ethical process. This article presents the study protocol of the EEC clinical trial: International Randomised Controlled Trial for the Treatment of Newly Diagnosed Ewing Sarcoma Family of Tumours (EURO EWING 2012).

## 2. Methods

### 2.1. Design and objectives

**Fig. 1:**
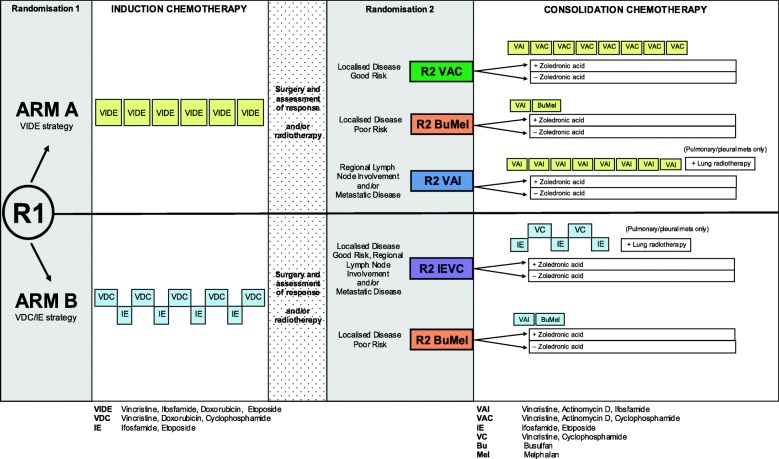
Trial schema

EURO EWING 2012 (EE2012) is an international, multicentre, phase III, open-label randomised controlled trial. There are two randomisations: patients are randomly assigned at entry (randomisation 1, or R1) and then following local control therapy (randomisation 2, or R2). The trial schema is shown in Fig. 1.

The objective of the induction/consolidation chemotherapy R1 is to compare

VIDE as induction chemotherapy and VAI/VAC/BuMel as consolidation chemotherapy (arm A) with VDC/IE induction and IE/VC or VAI/BuMel consolidation chemotherapy (arm B) as first-line treatment in all patients with ESFT with respect to clinical outcome and toxicity.

The objective of the zoledronic acid randomisation (R2) is to determine whether the addition of zoledronic acid to the consolidation chemotherapy assigned at R1 is associated with improved clinical outcome in patients in the EE2012 trial.

The third objective is to identify, through the biological studies embedded in EE2012, informative prognostic biomarkers for assessment of disease status and response at diagnosis and throughout the disease course. Whether they are predictive of response to therapy and may be used to improve stratification of patients and whether they might predict those patients who may not tolerate a particular therapy will also be explored.

### 2.2. Outcome measures

The primary outcome measure is EFS. EFS is defined as the time from randomisation to first event, where an event is progression without complete remission, recurrence (following complete or partial remission), second malignancy or death. Patients who do not have an event by the end of the follow-up period will be censored at their last follow-up date, and patients lost to follow-up without an event will be censored at the date of their last consultation.

The secondary outcome measures are the following:

- OS is defined as the time from randomisation to death, irrespective of the cause. Surviving patients will be censored at their last follow-up date.
- Adverse events and toxicity, defined by National Cancer Institute (NCI) Common Terminology Criteria for Adverse Events (CTCAE) version 4.0
- Histological response of the primary tumour to induction chemotherapy if surgery is performed as local control defined as the percentage of viable tumour cells in the resected primary tumour specimen
- Response of the primary tumour, regional lymph nodes and/or metastases, using volume of the whole primary tumour, diameter of the largest node (or group if not separate), and number of lung and/or pleural and other metastases respectively
- Achievement of local control at the end of treatment as defined by complete surgical resection following induction chemotherapy, no measurable disease as assessed by end-of-treatment magnetic resonance imaging (MRI) or computed tomography (CT) scan or no increase in measurable residual tumour over a 6-month period from the end of treatment
- Growth parameters and jaw/ear osteonecrosis (R2 only) will be assessed by using patient’s height measured at baseline, treatment and throughout follow-up for all patients who enter the second randomisation and who are younger than 18 years of age at entry. Whether jaw and ear osteonecrosis occurred will be recorded at the end of or during treatment for all patients who were randomly assigned to R2.

Primary tumour volume is assessed by using the following formula: tumour volume = a × b × c × F, where a, b and c represent the maximum tumour dimensions (in centimetres) in three planes; F = 0.52 for spherical tumours or F = 0.785 for cylindrical tumours.

If a pleural effusion is present (with a non-chest wall primary tumour), it is recorded along with the number of pulmonary metastases. For chest wall primary tumour, pleural effusion is considered loco-regional extension.

### 2.3. Recruitment and randomisation

All eligible patients with ESFT at participating centres are invited to take part in the trial. EEC partner organisations act as national coordinating centres (NCCs) and identify participating centres within their country or countries. The University of Birmingham is the coordinating sponsor and also undertakes the responsibilities of NCC in the UK. One hundred and ten participating centres are taking part across 10 countries (Belgium, the Czech Republic, Denmark, France, Hungary, Ireland, the Netherlands, Spain, Switzerland and the UK). Patients enter the trial via R1 and, if following induction chemotherapy they fulfil the R2 eligibility criteria, are asked to participate in R2.

Patients are eligible if all of the trial inclusion criteria are met and none of the exclusion criteria applies. The eligibility criteria originally excluded patients with extrapulmonary metastatic disease but this was amended in protocol version 3.0 in September 2016. (The exact date of implementation of this in each country varies as it was dependent upon gaining country-specific regulatory approvals.) The eligibility criteria for R1 and R2 are shown in Table 1.

**Table 1:** Inclusion and exclusion criteria

Randomisation 1	Randomisation 2
Inclusion criteria	
• Any histologically and genetically confirmed Ewing sarcoma family of tumours (ESFT) of bone or soft tissue, or round cell sarcomas ‘Ewing’s-like’ but negative for *EWSR1* gene rearrangement (Prior to protocol version 3.0, this read as ‘Histologically confirmed ESFT of bone or soft tissue, and Localised or pulmonary and/or pleural metastatic disease’.) • Age of more than 2 years and less than 50 years • Randomisation not more than 45 days after diagnostic biopsy/surgery • Patient medically fit to receive trial treatment • No prior treatment other than surgery	• Localised tumour **OR** metastatic disease and/or regional lymph node(s) involvement only at diagnosis and at least partial response of metastases and/or regional lymph node(s) (Prior to protocol version 3.0, this read as ‘Localised tumour **OR** Pulmonary and/or pleural metastatic disease only at diagnosis and at least partial response of the lung metastases and no progression of the primary tumour during induction chemotherapy’.) • Age of more than 5 years • Consolidation chemotherapy as per protocol intended • Medically fit to receive zoledronic acid
Exclusion criteria	
• Contra-indication to the treatment in randomisation 1 (R1) • Second malignancy • Pregnant or breastfeeding women	• History of dental surgery 6 months preceding the start of zoledronic acid or planned dental surgery during treatment or within 6 months after the end of treatment • History of jaw fracture • Ewing’s tumour of the maxilla or of the mandible • Progression of the primary tumour or appearance of new lesions

For each randomisation, patients are allocated in a 1:1 ratio to the two arms. Randomisation is performed by staff at participating centres online by using the randomisation function of the electronic remote data capture (eRDC) system designed and maintained by the coordinating sponsor.

The R1 randomisation is stratified by age at randomisation (<14 years or ≥14 years), sex, disease type (absence of metastases or involvement of lymph nodes only; lung or pleural metastases only; other metastases), volume of tumour at diagnosis (<200 mL or ≥200 mL) and country (the UK, France or other) to ensure that there is a balance between treatments within the strata defined by these key prognostic factors.

The R2 randomisation is stratified by allocated treatment in R1, age at R1 randomisation (<14 years or ≥14 years), sex, disease status (localised disease or regional lymph node involvement of lymph nodes only at diagnosis and good risk after induction, localised disease or regional lymph node involvement only at diagnosis and of lymph nodes only poor risk after induction, lung or pleural metastases at diagnosis, other metastasis at diagnosis), and country (the UK, France or other).

### 2.4. Trial treatment

**Randomisation R1:** At trial entry, patients are randomly assigned to one of the following treatment arms:

- Arm A (VIDE strategy): VIDE induction, VAI/VAC/BuMel consolidation
  Induction chemotherapy: six cycles of VIDE

Consolidation chemotherapy: one cycle of VAI plus seven cycles of VAC

(good risk localised disease) - R2 VAC.

**OR**

One cycle of VAI plus one cycle of BuMel (poor risk localised disease without contraindication to BuMel)

**OR**

Eight cycles of VAI (poor risk localised disease with contraindication to BuMel and/or regional lymph node(s) involvement and/or metastatic disease) - R2 VAI.

**OR**

- Arm B (VDC/IE strategy): VDC/IE induction, IE/VC/VAI/BuMel consolidation

Induction chemotherapy: nine cycles of alternating VDC and IE.

Consolidation chemotherapy: five cycles of alternating IE and VC - R2 IE/VC (good risk localised disease and/or regional lymph node(s) involvement and/or metastatic disease or poor risk localised disease with contraindication to BuMel.

**OR**

One cycle VAI plus BuMel (poor risk localised disease without contraindication to BuMel).

**Randomisation R2:** Following induction chemotherapy, patients who fulfil the eligibility criteria for R2 and consent to take part in the randomisation will receive consolidation chemotherapy as allocated at trial entry and be randomly assigned to receive either:

- **Nine cycles of zoledronic acid** following the first cycle of consolidation chemotherapy

**OR**

- No zoledronic acid

**Fig. 2:**
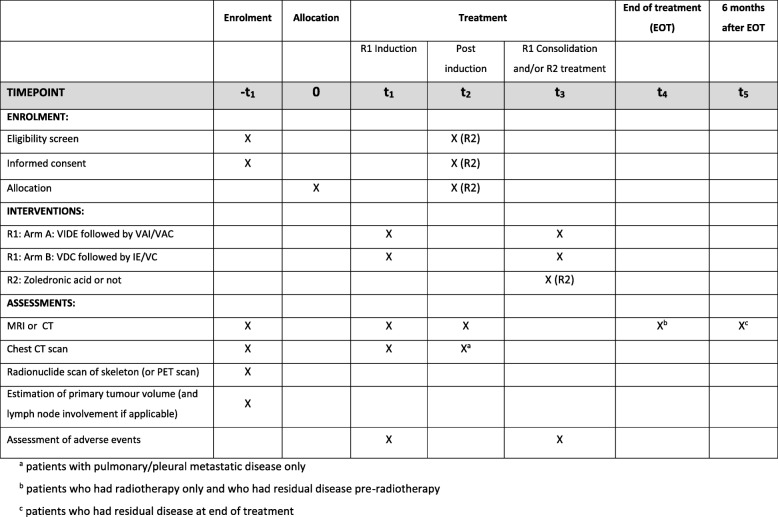
Summary schedule of enrolment, interventions and assessments

A summary of the enrolment, interventions and the main assessments is shown in Fig. 2, and a SPIRIT (Standard Protocol Items: Recommendations for Interventional Trials) checklist is supplied as Additional file 1. The full schedule of treatments is provided in Additional file 2: Table S1.

For good risk localised disease patients in arm A, a pragmatic decision was made to give VAC chemotherapy, as it is less toxic and requires less time in hospital and is equal to VAI in terms of outcomes. Local treatment of surgery or radiotherapy (or both) follows VIDE or VDC/IE induction chemotherapy, and whenever feasible, surgery proceeds after cycle 6 of VIDE (arm A) or cycle 9 of VDC/IE (arm B) on haematological recovery. Decisions on treatment of the primary tumour are individualised as is necessary in this disease. Consolidation chemotherapy is administered according to the treatment arm randomly assigned to and whether the patient has regional lymph node involvement or metastatic disease, or risk group for localised disease (good risk or poor risk). The definition localised poor risk and good risk is based on the presence or absence of a combination of factors, whether there is resection at diagnosis, tumour volume of at least 200 mL, pre-operative radiotherapy, histological response (≥10% viable tumour), unresectable tumour treated with radiotherapy alone, and volume of less than 200 mL with poor radiological response (Table 2).

**Table 2:** Definition of poor risk localised disease and indications for busulfan and melphalan (BuMel) high-dose therapy

Case	Localised disease	Resected at diagnosis	Volume ≥200 mL	Pre-operative RT	Histological response ≥10% viable tumour	Unresectable tumour treated with RT alone	Volume <200 mL but poor radiological response i.e., <50% regression with chemotherapy	RT contraindications to BuMel^a^	Other medical contraindications to BuMel^a^	BuMel recommended
1	N	n/r	n/r	n/r	n/r	n/r	n/r	n/r	n/r	N
2	Y	Y	N	n/r	n/r	n/r	n/r	n/r	n/r	N
3	Y	Y	Y	n/r	n/r	n/r	n/r	N	N	Y
4	Y	N	N	n/r	Y	n/r	n/r	N	N	Y
5	Y	N	N	Y	Y	n/r	n/r	N	N	Y
6	Y	N	Y	N	Y	n/r	n/r	N	N	Y
7	Y	N	Y	Y	Y	n/r	n/r	N	N	Y
8	Y	N	Y	Y	N	n/r	n/r	N	N	Y
9	Y	N	Y	Y	n/a^b^	n/r	n/r	N	N	Y
10	Y	N	Y	N	n/a	Y	n/r	N	N	Y
11	Y	N	N	N	n/a	Y	Y	N	N	Y

*n/a* not available, *n/r* not relevant, *RT* radiotherapy

^a^If response is yes, then high-dose therapy is contraindicated

^b^For example, if extracorporeal irradiation of primary tumour is used prior to re-implantation

Peripheral blood stem cell (PBSC) mobilisation and harvesting are recommended after VIDE/VDC/IE chemotherapy if defined as poor risk localised disease. PBSC mobilisation and harvesting should be performed in accordance with institutional guidelines. BuMel treatment is contraindicated for patients where radiotherapy is required to the central axial sites (spine, sacrum or pelvis) or when lung or bowel is within the radiotherapy treatment fields. (The protocol includes specific criteria regarding doses.) Radiotherapy is recommended to be given concurrently with consolidation chemotherapy to the primary site. In patients with pulmonary or pleural metastatic disease (or both), whole lung radiotherapy is recommended to be given on completion of consolidation chemotherapy. Radiotherapy to bony metastases may be given either during consolidation or at the end. At the end of treatment, an MRI or CT scan should be performed for patients who received radiotherapy only as local control and who had residual disease pre-radiotherapy. If the end-of-treatment scan shows residual disease, another scan should be performed 6 months after the end of treatment. After treatment, patients will be followed up with clinical evaluation and scanning for a minimum of 5 years or until disease progression or death if sooner. Patient data are collected on the eRDC by using a series of case report forms, and follow-up forms are requested annually following the completion of treatment in order to track patient status.

Patients are also asked to optionally consent to additional biological studies. Participation involves donating blood samples at multiple time points throughout the trial and agreeing to any remaining bone marrow and diagnostic tumour biopsy tissue taken as routine practise for research purposes.

### 2.5. Statistical considerations

#### 2.5.1. Randomisation 1

Owing to the rarity of ESFT and a restricted ability to randomly assign sufficient numbers of patients for a conventional design (with two-sided alpha = 0.05 and power = 80%), a Bayesian approach has been taken to the analysis of R1 which makes no prior assumptions that one chemotherapy arm is likely to be better than the other.

With a 5-year accrual period, it should be possible to randomly assign at least 600 patients across participating countries. Hence, the minimum sample size is set at 600. With a minimum of 2 years’ and a maximum of 7 years’ follow-up, there should be at least 150 events.

Non-informative priors will be used, so the posterior distribution gives Pr (parameter|data) (i.e., the probability of the treatment effect). The ln (hazard ratio or HR) is assumed to be normally distributed with variance 4/n, where n is the total number of events in both arms [16]. Based on the EURO-E.W.I.N.G. 99 data, 3-year EFS is anticipated to be about 70% with VIDE. Table 3 shows, for 600 patients, the probability that one treatment is better than the other, or not more than 5% worse, for a range of observed HRs. (An HR of 1.21, or inversely 0.81, represents about a 5% absolute difference in 3-year EFS.)

**Table 3:** Probability of one treatment being better

Observed 3-year EFS			Observed HR			*P* (HR <1.00)	*P* (HR <0.81)	*P* (HR >1.21)
VIDE	Difference	VDC/IE	Number of events	HR	ln (HR)			
0.70	0.00	0.70	180	1.00	0.00	0.50	0.07	0.10
0.70	0.05	0.75	165	0.81	−0.21	0.92	0.50	0.00
0.70	−0.05	0.65	195	1.21	0.19	0.09	0.00	0.50
0.70	0.025	0.725	173	0.90	−0.10	0.75	0.23	0.03
0.70	− 0.025	0.675	188	1.10	0.10	0.25	0.02	0.27

*EFS* event-free survival, *HR* hazard ratio, *VDC/IE* vincristine, doxorubicin, cyclophosphamide, ifosfamide and etoposide, *VIDE* vincristine, ifosfamide, doxorubicin and etoposide

The following can be seen:

- With an observed HR of 1.00 (no apparent difference between the randomly assigned groups in terms of EFS), there would be probabilities of 10% or 7% that VDC/IE was actually more than 5% worse or better respectively than VIDE, with a cumulative probability of 17% (i.e., within the limits of clinical acceptability). It would then be reasonable to base the decision on which regimen is preferable on toxicity.
- With an observed HR of 0.81 (an observed improvement of about 5% in EFS with VDC/IE compared to VIDE), there would be an 8% probability that the apparently better regimen (i.e., VDC/IE) was actually worse (i.e., within the limits of clinical acceptability).
- With an observed HR of 0.90 (i.e., about a 2.5% absolute difference in EFS in favour of VDC/IE), there would be a probability of 25% that the apparently better regimen was actually worse and a probability of 3% that it was more than 5% worse (i.e., at the limit of clinical acceptability).

#### 2.5.2. Randomisation 2

The R2 target is a minimum of 300 patients. An analysis of R2 will also be carried out in conjunction with the German Ewing 2008 trial which will have a similar or greater number of patients, giving a total of about 600 patients. (It is estimated 300 will come from EE2012 and 300 from Ewing 2008.) It is anticipated that it will take at least 5 years to reach the accrual targets. Patients will be followed up for progression and death until all trial objectives have been met. The first main analysis will be performed once all patients have a minimum of 2 years’ follow-up. For each randomisation, the main analyses will be intention-to-treat with all patients analysed in the arm to which they were allocated at randomisation.

For R2, conventional statistical analyses will be performed: Kaplan–Meier life tables will be constructed for time-to-event data (with date of randomisation as reference time point) and arms will be compared by means of the log-rank test; continuous variables will be compared across arms by means of *t* tests or Wilcoxon tests as appropriate. Multivariable analysis using Cox regression will be used to adjust for baseline co-variates as appropriate. Heterogeneity of the treatment effect according to these factors will be evaluated. As well as by individual trial, analyses of the zoledronic acid randomisation will be performed on the total data set for the two trials combined (with stratification by trial).

## 3. Discussion

Internationally, the standard drug treatment of ESFT is not defined. This study aims to address this and establish a “standard regimen” of chemotherapy, taking into account both clinical outcomes (EFS and OS) and toxicity. This new standard regimen will form the backbone of future international studies in ESFT, enhancing opportunities for collaboration and thus hastening progress in improving outcomes from this rare disease. In addition, this study will provide evidence to establish whether add-on treatment with a novel agent in this disease setting, zoledronic acid, is of benefit for patients with ESFT. Owing to the rarity of the tumour and the need for timely answers, the study has a pragmatic statistical design, accepting that it is not possible to reach conventional levels of reliability within a reasonable time frame, and will recruit patients across several European countries.

Although these tumours are the second commonest malignant bone tumour in children, adolescents and young adults, they remain rare tumours (fewer than 70 cases per year in the UK and 100 in France) and hence any randomised trial must be international. Setting up and activating large international trials are complex processes, involving multiple institutions each with their own local practises, and require acquiring approvals from numerous regulatory bodies across the participating countries. However, it is anticipated that the knowledge, experience and relationships formed through activating EE2012 internationally will be of benefit to any future trials established by the EEC and lead to shorter trial set-up times and therefore quicker answers to important therapeutic questions.

### 3.1. Trial status

The trial is open and the first patient was entered in March 2014. At the time of manuscript submission (April 2019), 639 and 242 patients had been recruited into R1 into R2 respectively. The current version of the protocol is version 5.0, dated 2 June 2017.

## Supplementary information

**Additional file 1.** SPIRIT Checklist.

**Additional file 2:** **Table S1.** Summary of treatment details: the schedules of administration of cycles of chemotherapy and zoledronic acid.

## Data Availability

No data are presented in this manuscript. The materials described can be obtained by contacting the corresponding author.

## Abbreviations

BuMel: Busulfan and mephalan
CT: Computed tomography
EE2012: EURO EWING 2012
EEC: EURO EWING Consortium
EFS: Event-free survival
eRDC: Electronic remote data capture
ESFT: Ewing sarcoma family of tumours
HR: Hazard ratio
IE: Ifosfamide and etoposide
MRI: Magnetic resonance imaging
N-BP: Nitrogen-containing bisphosphonate
NCC: National coordinating centre
OS: Overall survival
PBSC: Peripheral blood stem cell
R1: Randomisation 1
R2: Randomisation 2
SMN: Second malignant neoplasm
VAC: Vincristine, actinomycin D and cyclophosphamide
VAI: Vincristine, actinomycin D and ifosfamide
VC: Vincristine and cyclophosphamide
VDC: Vincristine, doxorubicin, cyclophosphamide
VIDE: Vincristine, ifosfamide, doxorubicin and etoposide
