# Supplementary material for: International randomised controlled trial for the treatment of newly diagnosed EWING sarcoma family of tumours – EURO EWING 2012 Protocol
Source: Trials. 2020 Jan 17;21:96. doi: 10.1186/s13063-019-4026-8 (PMC6969439; doi:10.1186/s13063-019-4026-8)
Supplement: Supplementary file 2 — Additional file 2: Table S1. Summary of treatment details: the schedules of administration of cycles of chemotherapy and zoledronic acid. [file 13063_2019_4026_MOESM2_ESM.docx]

Supplementary table 1: Summary of treatment

VIDE chemotherapy

Cycles of VIDE should be given at 21 day intervals (+/- 3 days) and on haematological recovery to absolute neutrophil count (ANC) ≥1.0x10^9^/L, platelets ≥80x10^9^/L.

| **VIDE** |  |  |  |  |
| --- | --- | --- | --- | --- |
| **V**INCRISTINE | 1.5 mg/m^2^  (IV push or short infusion) | d1 | (1.5 mg/m^2^/cycle) | *(max. single dose: 2 mg)* |
| **I**FOSFAMIDE | 3 g/m^2^/d  (IV infusion, 1-3 h) | d1, d2, d3 | (9 g/m^2^/cycle) | plus MESNA and hydration* |
| **D**OXORUBICIN | 20 mg/m^2^/d  (IV infusion, 4 h) | d1, d2, d3 | (60 mg/m^2^/cycle) |  |
| **E**TOPOSIDE  (etopophos can be used) | 150 mg/m^2^/d  (IV infusion, 2 h) | d1, d2, d3 | (450 mg/m^2^/cycle) |  |
| Granulocyte-colony stimulating factor (G-CSF) | | | | |
| *MESNA and hydration should be given according to institutional guidelines | | | | |

Patients whose surface area (SA) is > 2 m^2^ should have their doses capped and calculated with a SA of 2 m^2^.

VAI chemotherapy

Cycles of VAI should be given at 21 day intervals (+/- 3 days) and on haematological recovery to ANC ≥1.0x10^9^/L, platelets ≥80x10^9^/L.

| **VAI** |  |  |  |  |
| --- | --- | --- | --- | --- |
| **V**INCRISTINE | 1.5 mg/m^2^  (IV push or short infusion) | d1 | (1.5 mg/m^2^/cycle) | *(max. single dose: 2 mg)* |
| **A**CTINOMYCIN D | 0.75 mg/m^2^/d  (IV push) | d1, d2 | (1.5 mg/m^2^/cycle) | *(max. single dose per day: 1.5 mg)* |
| **I**FOSFAMIDE | 3 g/m^2^/d  (IV infusion, 1-3 h) | d1, d2 | (6 g/m^2^/cycle) | plus MESNA and hydration* |
| G-CSF |  | | | |
| *MESNA and hydration should be given according to institutional guidelines | | | | |

Patients whose SA is > 2 m^2^ should have their doses capped and calculated with a SA of 2 m^2^

VAC chemotherapy

Cycles of VAC should be given at 21 day intervals (+/- 3 days) and on haematological recovery to ANC ≥1.0x10^9^/L, platelets ≥80x10^9^/L.

| **VAC** |  |  |  |  |
| --- | --- | --- | --- | --- |
| **V**INCRISTINE | 1.5 mg/m^2^  (IV push or short infusion) | d1 | (1.5mg/m^2^/cycle) | *(max. single dose: 2 mg)* |
| **A**CTINOMYCIN D | 0.75 mg/m^2^/d  (IV push) | d1, d2 | (1.5mg/m^2^/cycle) | *(max. single dose per day: 1.5 mg)* |
| **C**YCLOPHOSPHAMIDE | 1500 mg/m^2^  (IV infusion, 1-3 h) | d1 | (1500mg/m^2^/cycle) | plus MESNA and hydration* |
| G-CSF |  | | | |
| *MESNA and hydration should be given according to institutional guidelines | | | | |

Patients whose SA is > 2 m^2^ should have their doses capped and calculated with a SA of 2 m^2^.

BuMel

BuMel should be given on haematological recovery to absolute neutrophil count (ANC) ³1.0x10^9^/L, platelets ³80x10^9^/L.

|  | Day | -7 | -6 | -5 | -4 | -3 | -2 | -1 | 0 |
| --- | --- | --- | --- | --- | --- | --- | --- | --- | --- |
| **Busulfan**, IV (total of 16 doses)  Adults:  0.8mg/kg. body weight (BW) | T = 0 |  | X | X | X | X |  |  |  |
| Children and adolescents:  <9kg: 1mg/kg. BW  9 - <16kg: 1.2mg/kg. BW | T = 6 | (X) | X | X | X | (X) |  |  |  |
| 16 - 23kg: 1.1mg/kg. BW  >23 - 34kg: = 0.95mg/kg. BW  >34kg: 0.8mg/kg. BW | T = 12 | X | X | X | X |  |  |  |  |
|  | T = 18 | X | X | X | X |  |  |  |  |
| **Melphalan**, IV  140mg/m² IV infusion over 30 min. |  |  |  |  |  |  | X |  |  |
| Clonazepam, orally or IV  0.025 to 0.1mg/kg/day |  | X | X | X | X | X | X | X |  |
| Stem cell re-infusion  (min. 3 x 10^6^/kg. CD34^+^) |  |  |  |  |  |  |  |  | X |

Hydration should be given according to institutional guidelines.

Patients whose SA is > 2 m2 should have their doses capped and calculated with a SA of 2 m2.

Heparin or allopurinol or ursodeoxycholic acid (UDCA) (Days -7 to +8) may be added according to institutional guidelines.

If the patient develops veno-occlusive disease, the management should be as institutional guidelines.

Zoledronic acid treatment

|  | Day 1 | Day 2 | Day 3 | Day 4 | Day 5 | Day 6 |
| --- | --- | --- | --- | --- | --- | --- |
| Prior to administering zoledronic acid, ensure adequate hydration of the patient in accordance with local practice  Normal creatinine clearance, electrolytes, calcium, magnesium, phosphate, bicarbonate, alkaline phosphatase | **X** |  |  |  |  |  |
| Zoledronic acid  0.05 mg/kg by IV infusion (maximum dose 4 mg). Patients 18 years or older will receive 4mg. The dose should be administered as an IV infusion over not less than 15 minutes. | **X** |  |  |  |  |  |
| Hydration 250ml/m^2^ (post zoledronic acid) | **X** |  |  |  |  |  |
| Oral calcium and vitamin D in accordance with local practice | **X** | **X** | **X** | **X** | **X** | **X** |
| Paracetamol in case of flu-like symptoms, in accordance with local practice | **X** | **X** | **X** | **X** | **X** | **X** |

VDC/IE chemotherapy

Alternating cycles of VDC and IE should be given at 14 day intervals (+/- 3 days) and on haematological recovery to absolute neutrophil count (ANC) ≥0.75x10^9^/L, platelets ≥75x10^9^/L. Blood counts should be obtained on day 7 and 14 of the cycle and every Monday, Wednesday, and Friday after Day 14, until the criteria for beginning the next cycle are satisfied.

| **VDC** | | | | |  |
| --- | --- | --- | --- | --- | --- |
| **V**INCRISTINE | 2 mg/m^2^  (IV push or short infusion) | d1 | (2 mg/m^2^/cycle) | *(max. single dose: 2 mg)* |  |
| **D**OXORUBICIN | 37.5 mg/m^2^/d  (IV infusion, 24 hrs) | d1, d2 | (75 mg/m^2^/cycle) |  |  |
| **C**YCLOPHOSPHAMIDE | 1200 mg/m^2^  (IV infusion, 1 hr) | d1 | (1200 mg/m^2^/cycle) | plus MESNA and hydration* |  |
| G-CSF |  | | | | |
| *MESNA and hydration should be given according to institutional guidelines | | | | |  |

| **IE** |  |  |  |  |
| --- | --- | --- | --- | --- |
| **I**FOSFAMIDE | 1800 mg/m^2^/d  (IV infusion, 1 h) | d1, d2, d3, d4, d5 | (9 g/m^2^/cycle) | plus MESNA and hydration* |
| **E**TOPOSIDE  (etopophos can  be used) | 100 mg/m^2^/d  (IV infusion, 2 h) | d1, d2, d3, d4, d5 | (500 mg/m^2^/cycle) |  |
| G-CSF |  | | | |
| *MESNA and hydration should be given according to institutional guidelines | | | | |

Patients whose SA is > 2m^2^ should have their doses capped and calculated with a SA of 2m^2^.

IE/VC chemotherapy

Alternating cycles of IE and VC should be given at 14 day intervals (+/- 3 days) and on haematological recovery to ANC ≥0.75x10^9^/L, platelets ≥75x10^9^/L. Blood counts should be obtained on day 7 and 14 of the cycle and every Monday, Wednesday, and Friday after Day 14, until the criteria for beginning the next cycle are satisfied.

| **IE** |  |  |  |  |
| --- | --- | --- | --- | --- |
| **I**FOSFAMIDE | 1800 mg/m^2^/d  (IV infusion, 1 h) | d1, d2, d3, d4, d5 | (9 g/m^2^/cycle) | plus MESNA and hydration* |
| **E**TOPOSIDE  (etopophos can  be used) | 100 mg/m^2^/d  (IV infusion, 2 h) | d1, d2, d3, d4, d5 | (500 mg/m^2^/cycle) |  |
| G-CSF |  | | | |
| *MESNA and hydration should be given according to institutional guidelines | | | | |

| **VC** |  |  |  |  |
| --- | --- | --- | --- | --- |
| **V**INCRISTINE | 2 mg/m^2^  (IV push or short infusion) | d1 | (2 mg/m^2^/cycle) | *(max. single dose: 2 mg)* |
| **C**YCLOPHOSPHAMIDE | 1200 mg/m^2^  (IV infusion, 1 hr) | d1 | (1200 mg/m^2^/cycle) | plus MESNA and hydration* |
| G-CSF |  | | | |
| *MESNA and hydration should be given according to institutional guidelines | | | | |

Patients whose SA is > 2 m^2^ should have their doses capped and calculated with a SA of 2 m^2^.
